# The Boston Medical Center Experience: An Achievable Model for the Delivery of Transgender Medical Care at an Academic Medical Center

**DOI:** 10.1089/trgh.2017.0054

**Published:** 2018-07-01

**Authors:** Pamela Klein, Supraja Narasimhan, Joshua D. Safer

**Affiliations:** ^1^ Boston Medical Center for Transgender Medicine and Surgery, Boston Medical Center and Boston University School of Medicine, Boston, Massachusetts.; ^2^ Boston Health Care for the Homeless Program, Boston, Massachusetts.; ^3^ Mount Sinai Center for Transgender Medicine and Surgery, Mount Sinai Health System and Icahn School of Medicine at Mount Sinai, New York, New York.

**Keywords:** academic medical centers, clinical care program design, transgender healthcare

## Abstract

Despite increasing appreciation for the medical needs of transgender individuals, the organization of transgender medical care remains suboptimal. Transgender individuals report difficulty in finding providers who have adequate expertise in caring for transgender patients, a lack of provider cultural competence, health system barriers, and discrimination in healthcare settings. At Boston Medical Center (BMC), we sought to address these gaps within an existing academic medical center. In February 2016, BMC established a Center for Transgender Medicine and Surgery (CTMS) to provide a single address for patients to obtain transgender-specific services across a spectrum of healthcare needs. With the establishment of a CTMS at BMC, we were able to leverage broad transgender medical coverage across multiple specialties within an existing academic medical framework. Furthermore, the development of the CTMS resulted in our identification of multiple gaps in transgender healthcare which we could target. Large gaps in care for our institution included genital surgery, perisurgical support, adolescent care, and care coordination. Notably, most of our interventions used existing resources. We propose that this is a replicable model that should be adopted by other academic medical institutions.

## Introduction

Transgender individuals have gender identities that differ from sex recorded at birth (typically determined by examination of external genitalia). In the United States, studies estimate that 0.6% of adults are transgender.^1^ The process of aligning physical characteristics with gender identity may involve any or all of the following: hormone therapy,^2^ surgery, change in appearance (e.g., with clothing, make-up, accessories), name change, change in identification documents, and/or change in pronoun use.^3^

As is well documented, transgender and gender-nonconforming individuals are much more likely to be poor or homeless than the average person. They are less likely to have gainful employment.^4–6^ Disparities in health outcomes for transgender and gender-nonconforming individuals are also well known and include elevated rates of anxiety, depression, suicide, attempted suicide, substance use, HIV, other sexually transmitted infections, and cigarette smoking. Transgender individuals are also disproportionately victims of violence compared with the general population.^7–9^

Boston Medical Center (BMC), in collaboration with Boston University School of Medicine (BUSM), was the first academic institution in the country to publish success with an evidence-based curriculum to teach transgender medicine to the future physician workforce.^10^ BMC has also been engaged in research activities to improve the healthcare of transgender patients.^11–14^

In 2016, to begin to further address the gap in access to care for transgender individuals, BMC established a Center for Transgender Medicine and Surgery (CTMS). This article describes the BMC model for a comprehensive program, along with lessons learned in our first year, to support our thesis that such an approach is a feasible, replicable framework to integrate transgender healthcare into an academic medical center.

## Program Description and First Year Observations

**FIG. 1.**
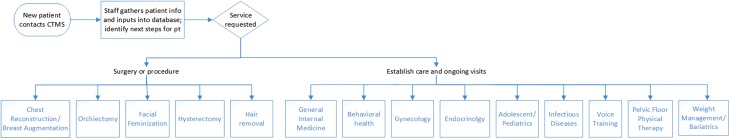
Process flow for coordinating patient care across BMC. This diagram outlines the services sought by patients and exemplifies the comprehensive nature of the CTMS. Once a patient contacts the CTMS and requested services are identified, a mechanism is in place to ensure proper navigation and coordination of those services. BMC, Boston Medical Center; CTMS, Center for Transgender Medicine and Surgery.

BMC created the CTMS to (1) assist patients in navigating the path to obtaining a neovaginoplasty, and (2) serve as a single point of contact for patients seeking transgender healthcare within the hospital. The initial administrative team included a physician medical director, a nurse liaison, and a project coordinator. The team created intake documents, a tracking system, and a process flow to move patients through the system (Fig. 1). The team followed the World Professional Association of Transgender Health (WPATH) Standards of Care to develop presurgical criteria.^15^ The team initiated collaborations with the clinical departments with which the patients would need to interact to fulfill presurgical requirements, and developed an approach for patients to have a single point of contact to meet all transgender medical care needs.

To educate staff members across different clinical areas about our new services, the team collaborated with staff and community members on the BMC Transgender Taskforce, an institution-wide organization developed to assure that transgender and gender-nonconforming individuals receive culturally competent care throughout the institution.

### Great heterogeneity in need was observed among patients seeking care

Although surgery was an early focus of the organized program, demand for other services was high and realized quickly. Indeed, the patients presenting to the CTMS were heterogeneous in their medical needs. Many made contact to obtain surgical procedures not previously accessible to them regionally, such as neovaginoplasty. Others sought nonsurgical transgender medical services, including hormone therapy, primary care, and mental healthcare. Still others had undergone surgery like neovaginoplasty elsewhere, and required revisions or other procedural follow-up. Patients were based locally and regionally. Patients requesting services ranged in age from 16 to 75 years.

### Great heterogeneity in previous healthcare experience was observed among patients seeking care

The range of patient experiences were broad. It was common that patients presented having primary care providers who were newly supportive based on recent publicity, but who lacked knowledge and confidence providing trans-specific care. Alternatively, patients presented from sophisticated existing transgender healthcare programs and were seeking only a defined specialized service like a specific surgery. Although a rarer event than observed historically, patients continued to present having only seen providers completely unsympathetic to transgender healthcare concerns.

### The heterogeneity among patients prompted the program to favor in-house surgical readiness assessment in lieu of outside letters of support

WPATH guidelines (and most insurers) require patients to provide two external behavioral health support letters.^15^ However, given the great heterogeneity in outside support, we often found in-house evaluation to be more useful to determine surgical readiness both from medical and psychosocial perspectives. Standard letters focused on diagnosis, but contained limited discussion surrounding medical morbidities. Outside letters did not provide sufficiently granular assessments of external supports necessary for healthy surgical outcomes. In practice, we found the transgender assessment of the patients was often clear without requiring the support letters. However, the medical clearance and the detailed navigation of external psychosocial issues proved the important areas requiring attention. Our protocol added a more involved in-house assessment of surgical readiness, which enabled us to better establish preparation for postsurgical self-care.

### The need for preoperative perineal hair removal was identified as a barrier to care

Like most surgeons, the BMC surgeons require an area of the perineum to be hair free before genital surgeries, such as vaginoplasty. With mainstream payers entering the equation, it became clear that a standard for most health insurance was noncoverage of all hair removal, classically considered “cosmetic.” The CTMS team needed to engage with policy makers to determine a strategy to provide coverage for the narrow instance of preoperative perineal hair removal before neovaginoplasty surgery, as the cost of this preoperative requirement could represent a significant barrier to care for some patients. Indeed, the cost of perineal hair removal can exceed the resources allowable for Medicaid-eligible patients.

### The need was recognized that facial feminization procedures can be medically necessary

While guidelines and insurance policies have tended to focus on genital surgeries as medically necessary with facial feminization surgeries referenced as cosmetic,^15^ it became clear that for some transgender women, facial feminization surgery is of greater consequence for physical alignment with gender identity and for safety relating to that physical alignment.^16^ The CTMS team was obligated to broach mechanisms to change policy so that the medical necessity of such interventions was addressed with clear criteria for coverage from payers.

### The need for patient input and peer support was identified

Patients sought peer support and the CTMS sought review of our program from patients who had healthcare experiences in our program. We initiated a patient support group for our genital surgery patients. The group offered the expected supportive environment to other patients. In addition, the group not only quickly became a much-needed source of patient feedback both for existing programmatic decisions but also for future programmatic priorities from a patient's perspective.

### The need for institutional education was identified

It became apparent that a large number of transgender individuals began to feel more empowered by the relative safety of the public embrace of transgender care by the institution. They swiftly reported the numerous gaps in transgender sensitivity beyond the areas that had been anticipated by our team. It was clear that it was insufficient to target education only to areas expected to play larger roles in transgender care, such as primary care, endocrinology, mental health, and plastic surgery. The only way to systematically address the educational needs of the institution was to initiate transgender medical and sensitivity education during new employee orientation and to identify ways to integrate this as part of ongoing employee evaluation.

### The need for adolescent care was identified

In Boston, like in most other locations in the United States, access to care for transgender youth has been even more limited than access for transgender adults. Boston is home to the original practice for support of transgender youth in the United States (the Gender Management Service or GeMS program at Boston Children's Hospital), but it soon became clear that the volume of need was far higher than could be supported by the existing local resources especially at the primary care level.

The BMC Department of Pediatrics worked together with the CTMS to create a program for youth (the Child and Adolescent Transgender Center for Health [CATCH] program) with the stated goal to provide support and care to children, adolescents, and young adults who identify as transgender, gender nonconforming, or are gender exploring and looking for additional support.

### Integrated professional and administrative coordination proved indispensable to program development

Because transgender care required coordination among multiple medical components of the institution along with numerous support services, the need for formal feedback loops swiftly became apparent. In practice, most of the feedback loops evolved organically when concerns or the recognition of potential concerns were recognized.

Within the first year of operation, the CTMS developed the following specific formal mechanisms for feedback: (1) The CTMS developed a forum for both patients and outside caregivers to provide feedback to the primary administration team through the main CTMS website. (2) The inpatient nursing team and the CTMS nurse liaison developed a formal communication avenue. (3) Patients awaiting surgery as well as patients who had completed surgery at BMC engaged in a monthly support group that also served as a safe forum for escalating communication to the CTMS on a defined, frequent, and ongoing basis.

CTMS surgical operations proved to be especially important elements of the program, where close coordination among patients, administration, providers, and support services required frequent, institution-customized adjustments. Early adjustments included: (1) Timing of some surgeries and length of in-hospital stay. (2) Preoperative criteria for some surgeries along with communication of such criteria. (3) Intrahospital and postdischarge pain protocols. (4) Postdischarge protocols in general for some surgeries.

### The need for external education was identified

The large gaps in care of transgender patients among medical providers was manifest in the Boston region with the result that BMC swiftly became a needed component for the dissemination of education to the regional healthcare community. Completely subscribed efforts included a day of transgender-related education that was open to local providers and a coordinated pelvic floor therapy in-service for area providers.

## Discussion

This is the first report of the potential to leverage an existing medical framework to optimize medical care for transgender individuals and substantially increase access to care. The strength of the program lies in the ability to constantly evolve and quickly adapt new processes and practices based on: (1) mechanisms to obtain feedback from patients, caregivers, and providers; (2) sharing feedback with the CTMS team in an efficient, timely manner through discussions at surgery readiness meetings; (3) absorbing best practices from other patient-centered BMC programs; and (4) working closely with the BMC Transgender Taskforce.

The maneuver also made the most efficient use of BMC resources by integrating transgender care into the existing framework of the institution. Patients needing behavioral health, endocrinology, primary care, including adolescent primary care, perineal hair depilation, gender-alignment surgery, or gynecology could call a single phone number and be connected to the appropriate service.

A broad institutional transgender taskforce proved instrumental to the success of the program. While the clinical program provided important services and provided the opportunity to leverage existing expertise, we found that the gap in competence to provide affirming care was large–even across a well-intentioned academic medical center. Training in clinical and culturally competent care had to be extended to all areas of the institution, including transport, pharmacy, radiology, emergency room, admitting, etc., and this proved a key symbiotic component of the overall programmatic success.

Collaboration among surgeons, CTMS staff, and inpatient nurses, along with feedback from patients and caregivers resulted in improvements in clinical care. Communication proved important with regular meetings indispensable in providing coordinated care.

In caring for transgender patients, departments within an academic medical institution can collaborate effectively. Although BMC has cared for transgender patients for many years, providing many transgender-specific medical and surgical services, including primary care, endocrinology, chest surgery, gynecological services, and facial feminization surgery, these services have always been provided within specific departmental “silos.” The CTMS unified transgender care across the departments and divisions of Primary Care, Endocrinology, Plastic Surgery, Urology, Dermatology, Ob/Gyn, Infectious Disease, ENT, and Behavioral Health under one umbrella, improving institution-wide collaboration in an efficient and patient-centered manner to improve patient care.

## Conclusion

With modest investment, we leveraged existing medical services to provide a unified, comprehensive program to provide transgender-related healthcare in an academic institution. The BMC model should be replicable and sustainable at other conventionally organized academic medical centers.

## Abbreviations Used

BMC: Boston Medical Center
CTMS: Center for Transgender Medicine and Surgery
WPATH: World Professional Association of Transgender Health
